# O.4.2-9 Shared genetic factors may partly explain the associations between physical activity and cardiometabolic diseases

**DOI:** 10.1093/eurpub/ckad133.184

**Published:** 2023-09-11

**Authors:** Elina Sillanpää, Niko Tynkkynen, Timo Törmäkangas, Teemu Palviainen, Matti Hyvärinen, Laura Joensuu, Marie Klevjer, Anja Bye, Pesonen Paula, Waller Katja, Kangas Maarit, Männikkö Minna, Vähä-Ypyä Henri, Sievänen Harri, Korpelainen Raija, Jämsä Timo, Niemelä Maisa, Ripatti Samuli, Kujala Urho, Kaprio Jaakko

**Affiliations:** Faculty of Sport and Health Sciences, University of Jyväskylä, Finland; Faculty of Sport and Health Sciences, University of Jyväskylä, Finland; Faculty of Sport and Health Sciences, University of Jyväskylä, Finland; Institute for Molecular Medicine Finland (FIMM), University of Helsinki, Finland; Faculty of Sport and Health Sciences, University of Jyväskylä, Finland; Faculty of Sport and Health Sciences, University of Jyväskylä, Finland; Department of Circulation and Medical Imaging, Faculty of Medicine and Health Sciences, Norwegian University of Science and Technology, Trondheim, Norway; Department of Circulation and Medical Imaging, Faculty of Medicine and Health Sciences, Norwegian University of Science and Technology, Trondheim, Norway; Northern Finland Birth Cohorts, Infrastructure for population studies, Faculty of Medicine, University of Oulu, Oulu, Finland; Faculty of Sport and Health Sciences, University of Jyväskylä, Finland; Research Unit of Medical Imaging, Physics and Technology, University of Oulu, Oulu, Finland; Research Unit of Medical Imaging, Physics and Technology, University of Oulu, Oulu, Finland; The UKK Institute for Health Promotion Research, Tampere, Finland; elina.sillanpaa@jyu.fi; The UKK Institute for Health Promotion Research, Tampere, Finland; elina.sillanpaa@jyu.fi; Northern Finland Birth Cohorts, Infrastructure for population studies, Faculty of Medicine, University of Oulu, Oulu, Finland; Research Unit of Medical Imaging, Physics and Technology, University of Oulu, Oulu, Finland; Research Unit of Medical Imaging, Physics and Technology, University of Oulu, Oulu, Finland; Institute for Molecular Medicine Finland (FIMM), University of Helsinki, Finland; Faculty of Sport and Health Sciences, University of Jyväskylä, Finland; Institute for Molecular Medicine Finland (FIMM), University of Helsinki, Finland

## Abstract

Shared genetic factors may contribute to the associations between higher levels of physical activity (PA) and lower risk for cardiometabolic diseases (CMDs), and may partially explain these associations observed in cohort studies. To explore this, we used novel methodology to calculate PA genotypes (polygenic risk score, PRS) and validated them against measured or reported PA in three independent cohorts. We then investigated the associations between polygenic inheritance of PA and cardiometabolic risk factors and diseases in two large population-based biobank datasets, and examined whether selected associations were independent of self-reported PA.

Our study utilized the UK Biobank as a base dataset (N = 400,124) and constructed genomewide PRSs for both self-reported and device-measured PA using single nucleotide polymorphism (SNP)-specific weights and SBayesR methodology. Both PRSs for PA included over one million SNPs. PRSs were constructed in the Finnish Twin cohort (N = 759–11,528), the Northern Finland Birth Cohort 1966 (N = 3,263–4,061), the Trøndelag Health Study cohort (HUNT, N = 47,148), and the FinnGen (N = 218,792). Cardiometabolic risk factors were measured in laboratory conditions, and CMD outcomes were derived from national health registers (ICD codes). We utilized linear, logistic, and cox regression methods for analysis.

Our results showed that genotypes predisposing to higher PA were associated with higher levels of PA in independent datasets, but PRSs accounted for only a limited amount of variation (0.13-1.44%). Genotypes supporting higher PA were associated with lower body mass index [B=-0.002 in HUNT and B=-0.025 in FinnGen] and favorable cardiometabolic health in HUNT (waist circumference [B=-0.003] and HDL cholesterol [B = 0.004]). Genotypes supporting higher PA volumes were associated with lower incidence of CMDs in both HUNT and FinnGen. The strongest associations were found in hypertensive diseases and Type 2 Diabetes. In HUNT, the observed associations were not materially changed after accounting for self-reported PA. Higher PRS for PA was also associated with lower risk of mortality in FinnGen.

Our findings suggest small pleiotropic effects between PA and CMDs. This means that same genetic variation may explain both physical activity behaviour and risk of diseases. PRSs provide new tools for genetic studies in sport science, but they currently have substantial practical limitations.

